# A model to forecast the two-year variation of subjective wellbeing in the elderly population

**DOI:** 10.1186/s12911-023-02360-8

**Published:** 2023-11-08

**Authors:** Isotta Trescato, Chiara Roversi, Martina Vettoretti, Barbara Di Camillo, Andrea Facchinetti

**Affiliations:** 1 Department of Information Engineering, University of Padova, Padova (PD), Italy; 2 Department of Comparative Biomedicine and Food Science, University of Padova, Legnaro (PD), Italy

**Keywords:** Wellbeing, CASP-12 score, Machine learning, Forecasting model, Ageing

## Abstract

**Background:**

The ageing global population presents significant public health challenges, especially in relation to the subjective wellbeing of the elderly. In this study, our aim was to investigate the potential for developing a model to forecast the two-year variation of the perceived wellbeing of individuals aged over 50. We also aimed to identify the variables that predict changes in subjective wellbeing, as measured by the CASP-12 scale, over a two-year period.

**Methods:**

Data from the European SHARE project were used, specifically the demographic, health, social and financial variables of 9422 subjects. The subjective wellbeing was measured through the CASP-12 scale. The study outcome was defined as binary, i.e., worsening/not worsening of the variation of CASP-12 in 2 years. Logistic regression, logistic regression with LASSO regularisation, and random forest were considered candidate models. Performance was assessed in terms of accuracy in correctly predicting the outcome, Area Under the Curve (AUC), and F1 score.

**Results:**

The best-performing model was the random forest, achieving an accuracy of 65%, AUC = 0.659, and F1 = 0.710. All models proved to be able to generalise both across subjects and over time. The most predictive variables were the CASP-12 score at baseline, the presence of depression and financial difficulties.

**Conclusions:**

While we identify the random forest model as the more suitable, given the similarity of performance, the models based on logistic regression or on logistic regression with LASSO regularisation are also possible options.

**Supplementary Information:**

The online version contains supplementary material available at 10.1186/s12911-023-02360-8.

## Background

Population ageing is a well-established phenomenon grounded on declining fertility and increased longevity: the demographic shift is evident at a global level, impacting the healthcare system, the social balance, and financial matters. The implications of this process are therefore significant for individuals, families, and governments, raising the interest of the research community. A constant effort is made to characterise and foster healthy ageing [1] because disabilities in later life are generally attributable to a combination of genetic, lifestyle, and environmental factors [2], and because older adults tend to have higher rates of severe chronic health issues.

In this context, the concept of Quality of Life (QoL) is widely studied, although not uniquely defined [3]. While it is still debated whether the QoL is better defined by the patient or the doctor, whether it is an objective or a subjective phenomenon and even if it can be measured at all, QoL is often considered a health-related measure. Some of the most common means of measuring wellbeing are the Health-Related Quality of Life (HRQoL) measures, commonly used in clinical practice, for example, the Quality of Adjusted Life Years (QALYs), in which the concept of the quantity of healthy years is assumed to be representative of the quality assessment [4].

With the growing belief that QoL should be considered a complex and differentiated phenomenon, accounting for people’s ability to overcome illness and adapt their lives to pursue their goals, Hyde, Wiggins, Higgs and Blane developed the CASP-19 score, a theoretically grounded measure, to overcome the limitations of the existing measures of QoL [5]. The CASP-19 score is specifically developed to investigate the perception of wellbeing in the elderly population, taking into account the peculiarities of that specific age range, characterised by the transition from work to retirement, and consequently by the variation in social dynamics [5]. In the original version of the score, 19 answers are collected through a Likert scale with four options: often, sometimes, not often, and never. An abbreviated version of the scale, CASP-12, was later proposed for the same purpose: for each question, a score between 1 and 4 is assigned: 1 for the response “often”, 2 for the response “sometimes”, 3 for the response “rarely” and 4 for the response “never”. The resulting score ranges between 12 and 48, with a higher score indicating a higher perceived wellbeing [6]. Recent work has focused on validating the CASP-12 scale to assess its internal consistency and reliability, establishing that CASP-12 can be effectively used as a multidimensional tool to assess wellbeing in older people [7–9].

Several studies suggested that a lower CASP-12 score may be connected with a higher risk of chronic diseases. Significant associations were found between wellbeing and incident arthritis [10], chronic obstructive pulmonary disease (COPD) risk [11], life expectancy (and healthy life expectancy) [12], and glycated haemoglobin levels [13]. Other works focused on discovering the variables that can most influence subjective wellbeing. In particular, cross-sectional studies investigating the relationship between the CASP-12 score and demographic, clinical, economic, and social variables have shown that CASP-12 score tends to decline after the age of 68 [6, 14] and that it is influenced by financial status and by the quality of social relationships. One relevant longitudinal study is the one from Webb et al. [15], where the authors demonstrated that improving health, perceived financial situation, increasing income, and frequency of contact with friends, are linked to an increase in CASP-12 score after four years. The main limitation of this study is that, since the input variables are used with their baseline value and also accounting for the difference between the value at baseline and the value at the time of the outcome, it should be considered a descriptive model rather than predictive. Additionally, the authors only report the performance of the model on the training set and do not validate it on any test set.

While the concept of QoL has been widely studied, and high perceived wellbeing can be crucial for promoting a healthy life among elderly individuals [16], the development of models to forecast CASP-12 variations has been relatively unexplored. This may be due either to a lack of suitable longitudinal datasets before the availability of the U.S. Health and Retirement Study and the English Longitudinal Study of Ageing (ELSA) and the Survey of Health, Ageing and Retirement in Europe (SHARE) projects, or because forecasting a complex measure such as perceived wellbeing can be extremely challenging since it might be influenced by numerous factors.

The aim of this work was to explore the possibility of creating a model to forecast the variation of CASP-12 score ahead of time using statistical/machine learning models fed by demographic, health, social and financial variables and, as a by-product, identify which variables are the most important in the prediction process. Specifically, the models we developed target a binary outcome, namely the 2-year variation of the CASP-12 score (worsening/not worsening). To achieve our goal, we leveraged data from SHARE, a longitudinal and publicly available dataset, which is described in detail in the following.

## Dataset and preprocessing

### The Survey of Health, Ageing and Retirement in Europe

The Survey of Health, Ageing and Retirement in Europe (SHARE) is a multidisciplinary longitudinal survey that investigates the social, economic, and health situation of community-dwelling individuals aged 50 and over in Europe [17]. SHARE is mainly funded by the European Commission (Horizon 2020), the US National Institute on Aging, and the German Federal Ministry of Education and Research.

The first wave dates back to 2004, and involved participants from 11 European countries. With an increasing number of countries and individuals joining the project, SHARE has completed eight waves of data collection, with the latest wave, involving more than 140000 participants, conducted in 2020. The data are gathered through face-to-face Computer-Aided Personal Interviews (CAPI) supplemented by a self-completion paper and pencil questionnaire [18]. Additionally, SHARE conducts end-of-life interviews for participants who decease. Apart from individuals aged 50 and over who have provided their written consent, SHARE also interviews their partners in all waves, regardless of age. The data collected across the SHARE waves measure physical and mental health, economic and non-economic activities, income and wealth, transfers of time and money within and outside the family, as well as life satisfaction and wellbeing [18]. The only exceptions pertain to the third and seventh waves, which are retrospective and aim to investigate people’s life histories. More in detail, Wave 3 is only retrospective while the Wave 7 questionnaire contains a retrospective questionnaire for all respondents who did not participate in Wave 3, as well as a regular panel questionnaire for all respondents who already answered Wave 3.

### Preprocessing

#### Selection of waves and subjects

As previously outlined, our study aimed at investigating the longitudinal variations of the CASP-12 score in elderly individuals. Due to the retrospective nature of the third wave of SHARE, which only collects variables on the participants’ life history, to maximise the number of consecutive assessments we opted to include only waves 4 to 7, collected in 2011, 2013, 2015, and 2017, in our analysis. It is relevant to mention that, despite being retrospective like wave 3, wave 7 was still valuable for our purposes as it includes responses to the CASP-12 questionnaire.

**Fig. 1:**
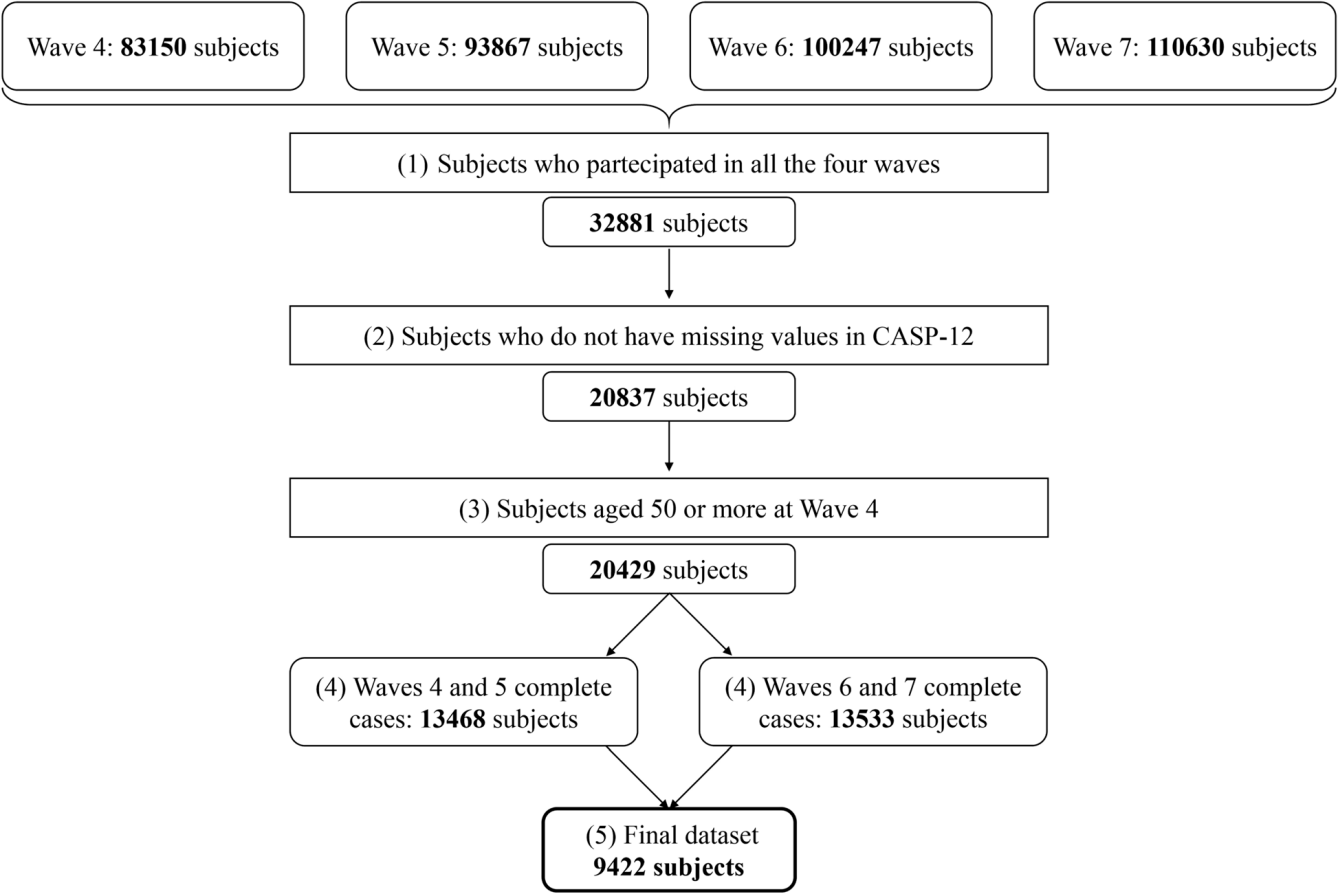
Flowchart of subject selection

Subsequently, we selected subjects who meet the following criteria: (1) participated in all four selected waves, to enable comparing the performance of the models over time on the same set of subjects, thus avoiding possible bias due to changes in the population (2) have no missing values in the CASP-12 score; (3) are aged 50 years old or older, to consider the target group of the SHARE study and to avoid potential biases of using CASP-12, which is specifically designed for the elderly, in an excessively young population. These selection steps are represented in Fig. 1 together with the number of subjects retained at each step.

#### Selection of variables

From all the available variables, a meaningful subset was selected following the most commonly used in literature [14, 15]. Table 1 reports a summary of the selected variables, including their names in the first column, brief descriptions in the second column, possible levels for categorical variables, and range of admitted values for continuous variables in the third column. In the fourth column, the distribution of the 20429 subjects who participated in all the considered waves, with complete information on CASP-12 scores, and who were aged more than 50 at the first wave is reported. In the fifth column, the distribution of the 13468 subjects having complete information for all the selected variables in the fourth wave is displayed. Finally, in the sixth column, the distribution of the 9422 subjects used to train the models is presented. For categorical variables the percentages of subjects for each level are reported, while for continuous variables, the median [1st quartile; 3rd quartile] is reported. The percentages in the fourth column are computed excluding missing values to enable comparison with the values in the fifth column.

**Table 1:** Summary of the selected variables, including names (first column), brief descriptions (second column), possible levels for categorical variables, and the range of admitted values for continuous variables (third column). In the fourth, fifth, and sixth columns, the distributions of the variables in the datasets for steps 3, 4, and 5 of Fig. 1 are reported

Variable	Description	Levels	Subjects aged 50 or more at Wave 4	waves 4 and 5 complete cases	Final selection
sample size			20429	13468	9422
Demographic					
age${}^{\star }$ (0%)	Age at baseline	>50	64 [58;71]	64 [58;71]	65 [58;72]
country${}^{\dagger \star }$ (0%)	Residence country	Austria	9.64	10.97	12.19
		Belgium	11.42	12.36	12.96
		Czech Republic	10.22	7.72	7.26
		Denmark	6.40	6.65	6.75
		Estonia	13.83	15.02	15.93
		France	9.42	10.04	9.89
		Germany	3.58	3.62	3.44
		Italy	8.22	7.86	7.02
		Slovenia	5.68	6.62	7.26
		Spain	7.84	7.42	6.18
		Sweden	4.47	4.67	4.87
		Switzerland	9.28	7.06	6.25
gender${}^{\star }$ (0%)	Gender	female	58.22	59.21	64.04
		male	41.78	40.79	35.96
ISCED (1.16%)	Instruction level, according to ISCED 97	0	2.28	2.37	2.29
		1	16.23	15.85	15.58
		2	17.71	17.05	16.95
		3	35.15	35.11	35.05
		4	5.43	5.37	5.39
		5	22.38	23.34	23.68
		6	0.82	0.91	1.06
marital status${}^{\dagger \star }$ (0%)	Marital status	living alone	23.66	31.50	41.04
		living together	70.42	60.73	48.97
		never married	5.92	7.77	9.99
Health-related					
ADL (0.01%)	Limitations in Activities of Daily Living	0	92.30	91.84	91.56
		1	4.91	5.14	5.37
		2	1.41	1.57	1.63
		3	0.71	0.77	0.72
		4	0.26	0.32	0.34
		5	0.24	0.22	0.24
		6	0.18	0.13	0.13
CASP-12 (0%)	CASP-12 score	[12, 48]	39 [34; 43]	39 [34; 43]	39 [34; 42]
chronic diseases (0.02%)	Number of chronic diseases	0	37.01	36.15	35.48
		1	31.36	31.54	31.67
		2	18.64	18.67	18.85
		3	8.64	9.02	9.14
		4	2.91	3.13	3.26
		5	1.09	1.10	1.20
		6	0.27	0.29	0.33
		7	0.08	0.08	0.06
		8	0.01	0.01	0.01
EURO D${}^{\dagger \star }$ (0 %)	Depression score	0	21.49	20.57	19.52
		1	22.51	22.07	21.79
		2	17.82	17.84	17.58
		3	13.23	13.24	13.60
		4	9.94	10.36	10.92
		5	6.51	6.77	6.75
		6	3.55	3.66	3.96
		7	2.54	2.75	3.02
		8	1.15	1.36	1.45
		9	0.74	0.08	0.80
		10	0.36	0.39	0.41
		11	0.13	0.16	0.17
		12	0.03	0.04	0.03
health (0 %)	Self assessment of general health	1	8.16	8.31	8.41
		2	19.81	19.31	19.09
		3	37.59	37.15	36.03
		4	26.88	27.41	28.27
		5	7.55	7.82	8.19
IADL (0.01%)	Limitations in Instrumental ADL	0	88.25	87.78	87.13
		1	7.95	8.20	8.63
		2	2.25	2.44	2.55
		3	0.78	0.84	0.91
		4	0.36	0.35	0.38
		5	0.20	0.21	0.20
		6	0.13	0.12	0.14
		7	0.08	0.07	0.06
mobility (0%)	Number of mobility limitations	0	53.83	52.70	51.36
		1	16.26	16.43	16.27
		2	10.04	10.27	10.43
		3	6.51	6.36	6.76
		4	4.55	4.70	5.06
		5	3.06	3.37	3.49
		6	2.30	2.52	2.79
		7	1.60	1.75	1.90
		8	1.02	1.03	1.03
		9	0.50	0.59	0.63
		10	0.31	0.27	0.28
moderate act (0.29%)	Frequency of moderate phisical activity, 1=more than once a week; 4=hardly ever or never	1	73.49	73.44	73.17
		2	13.39	13.41	13.48
		3	5.33	5.35	5.35
		4	7.79	7.80	8.00
vigorous act (0.28%)	Frequency of vigorous phisical activity, 1= more than once a week; 4=hardly ever or never	1	36.81	36.55	35.62
		2	15.29	14.96	14.89
		3	9.65	9.67	9.50
		4	38.25	38.81	39.99
Social					
left out (0%)	Feeling of being left out, 1=often; 4=never	1	5.98	6.18	6.55
		2	19.07	18.94	19.28
		3	27.19	26.94	26.59
		4	47.77	47.94	47.58
social activities (0.29%)	1.3cmNumber of social activities	0	11.80	11.62	11.05
		1	20.68	20.55	20.05
		2	24.89	24.72	24.43
		3	21.57	21.83	22.82
		4	12.93	13.08	13.45
		5	5.90	6.03	6.10
		6	1.88	1.80	1.77
		7	0.36	0.37	0.33
Financial					
cars${}^{\star }$ (28.61%)	Number of owned cars	0	27.29	26.89	31.61
		1	53.35	53.51	52.24
		2	19.36	19.59	16.15
current job (0.28%)	Current job status	employed	30.18	29.94	29.31
		retired	62.93	62.90	63.37
		unemployed	6.89	7.16	7.31
ends meet (28.84%)	Difficulties in making ends meet,0=with great difficulty; 4=easily	1	9.20	9.40	10.10
		2	26.21	26.14	26.85
		3	34.00	34.21	33.97
		4	30.59	30.25	29.07
owner occupier${}^{\star }$ (28.77%)	Being an owner-occupier	not owner	24.52	23.91	26.84
		owner	75.48	76.09	73.16

Categorical variables include percentages of subjects, while continuous variables are reported as the median [1$^{st}$ quartile; 3$^{rd}$ quartile], for steps 3, 4, and 5 of the flowchart. Missing value percentages for each variable in Wave 4 are provided below the variables’ names. $\dagger$ denotes the variables statistically different between the fourth and the fifth columns, $\star$ those different between the fifth and the sixth columns

To assess the potential bias introduced with the selection process, two different statistical tests were performed, depending on the variables’ type. The chi-squared test was applied for continuous variables and the Wilcoxon test for the categorical ones. In Table 1, variables with a statistically relevant difference between the first two datasets (points 3 and 4 of Fig. 1) are highlighted with a $\dagger$ symbol, while those with a statistically significant difference between points 4 and 5 of Fig. 1 are denoted by a $\star$ symbol.

The selected variables that accounted for demographic data were age, gender, education and marital status. Other variables were related to the health status of the subjects: ADL and IADL, two scales that measure mobility limitations in Activities of Daily Living and in the Instrumental Activities of Daily Living, respectively; the number of chronic diseases ever diagnosed; EURO-D, a scale used to assess the severity of depression; a self-evaluated score on the health status; the total number of mobility limitations; the frequency of moderate and vigorous physical activity. The social sphere was considered by including the feeling of being left out and a variable counting the number of social activities, such as volunteering or attending a social club, in which each subject is involved. Moreover, variables related to the economic situation were included: the number of owned cars, the current job situation, a subjective evaluation of the difficulty in making ends meet, and the ownership of the house.

#### Definition of the outcome

A binary variable representing the worsening or increasing of subjects’ wellbeing in two subsequent waves was considered as the outcome of the model. In detail, we first considered two subsequent waves among the four selected and assigned the first one as the baseline and the second one as the follow-up. We then computed the $\Delta {\text {CASP-12}}$ as the difference of the score between the follow-up wave ($t_1$) and the baseline wave ($t_0$):

1 $$\begin{aligned} \Delta {\text {CASP-12}}={\text {CASP-12}}_{t_1}-{\text {CASP-12}}_{t_0} \end{aligned}$$

The outcome variable *y* was defined as follow:

2 $$\begin{aligned} y= \left\{ \begin{array}{rl} 1 \qquad if \; \Delta {\text {CASP-12}} <0 \\ 0 \qquad if \; \Delta {\text {CASP-12}} \ge 0 \end{array} \right. \end{aligned}$$

thus being equal to 1 if the CASP-12 score decreases, while equal to 0 if it increases or remains constant in the 2 waves. This definition of the outcome allowed us to predict a longitudinal variation of the CASP-12 score with a prediction horizon of 2 years.

#### Creation of the training and the test set

A second step of subject selection was then performed, to include only complete cases of all variables in all the selected waves (i.e., the subjects without missing values in any of the considered variables). The available data were then split into a training set, containing 80% of randomly chosen subjects, and an independent test set containing the remaining 20% of subjects. Splitting of subjects was performed stratifying by age and CASP-12 score at baseline. Finally, to facilitate the comparison of the model coefficients, all the numerical predictor variables were scaled by their maximum value, to fit in a range between 0 and 1. The normalisation parameters, for both the training and the test set, were calculated on the training set.

## Method

Three different techniques were employed to develop models for forecasting the CASP-12 score variation two years ahead: two are linear models, namely the logistic regression (LR) and the logistic regression with Least Absolute Shrinkage and Selection Operator (LASSO) regularisation (see Appendix I, Additional file 1); the last is a non-linear technique, the random forest (RF), (see Appendix II, Additional file 1). As detailed in Appendix I and II, we employed 10-fold cross-validation to tune the model hyperparameters for LASSO and RF. Specifically, the optimal value of the regularisation parameter $\lambda$ in LASSO regularisation was determined by searching a range of values between $\lambda _{min}$ and $\lambda _{max}$, and selecting the value that produced the smallest deviance on 10-fold cross-validation [19]. The optimal threshold for the class assignment was set equal to the probability value corresponding to the closest point to the upper left corner of the ROC curve. For the RF model, we tested the number of trees between 500 and 1000 and the number of variables considered at each split between 4 and 8, and selected the combination that resulted in the best accuracy value during cross-validation.

Waves 4 and 5 were considered for the development of the models, thus using the predictors at wave 4, considered as the baseline, to forecast the CASP-12 score variation between waves 4 and 5. The models’ performance was evaluated as described in Assessment of model performance section. Assessment of models’ generalisability and stability section describes how we investigated the generalisability of the models using different subsets of subjects for training and testing the models, the possibility of applying the models to future waves, and the stability of the most important predictors in different waves.

In addition to developing a forecasting model of wellbeing, we are also interested in identifying the strongest predictors of wellbeing. For this purpose, we considered the variables maintained by LASSO and those with greater absolute values of the model coefficients to be more relevant, while for RF models we relied on the variable importance as determined by the mean decrease in Gini index [20].

### Assessment of model performance

To assess the forecasting performance of the developed models, we used four specific metrics for binary predictions: accuracy, Receiver Operating Characteristic (ROC) curve, Area under the ROC Curve (AUC), and F1 score.

In binary prediction, comparing the predicted values with the true values, four cases can be identified:

- True positive (TP): the number of observations for which the event occurs and the model predicts it correctly;
- False positive (FP): the number of observations for which the event does not occur, but the model predicts its occurrence (also known as type I error);
- True negative (TN): the number of observations for which the event does not occur and the model predicts it correctly;
- False negative (FN): the number of observations for which the event occurs, but the model predicts it does not occur (also known as type II error).

Accuracy is defined as the number of correctly predicted observations out of all the data points, formally:

3 $$\begin{aligned} accuracy=\frac{TP+TN}{TP+FP+TN+FN} \end{aligned}$$

Being True Positive Rate defined as $TPR=\frac{TP}{TP+FN}$ and False Positive Rate as $FPR=\frac{FP}{TN+FP}$, the ROC (Receiver Operating Characteristic) curve is plotted with TPR (on the x-axis) against the FPR (on the y-axis), for different values of the classification threshold. AUC represents a measure of the separability of classes, in a range of [0, 1]. An AUC value of 0.5 means that the model is not better than a classifier that randomly assigns the classes, while a value of 1 corresponds to the perfect model that perfectly separates the classes. The F1 score is defined as the harmonic mean of precision and recall, where precision is the number of TP divided by $TP+FP$, and recall is the number of TP divided by the sum of $TP+FN$. The F1 score provides a single metric that balances precision and recall and is useful to evaluate the overall performance of a binary prediction model. The F1 score ranges in [0, 1], where 0 indicates poor precision and recall and 1 indicates perfect precision and recall.

### Assessment of models’ generalisability and stability

#### Assessment of model’s generalisability on different subjects

To assess the robustness of the models, each of them was trained and tested on ten different training-testing splits. Each training-testing split was created as specified in Creation of the training and the test set section. For each split, the hyperparameters of each model were tuned on the training set. The mean and the standard deviation (SD) of Accuracy, AUC, and F1 score on the ten testing splits are then calculated. In particular, the SD is a key indicator to evaluate that subjects splitting into training/test sets does not introduce a relevant bias (the lower the SD, the more robust the model).

#### Assessment of models’ stability over time

The longitudinal nature of the SHARE dataset allowed us to verify the stability of the models over time. The original models were trained using predictor values at wave 4, with a binary outcome based on the change in the CASP-12 score between wave 4 and 5. To test the models’ stability, we extracted the variables of interest from two later waves (waves 6 and 7) while keeping the pool of subjects in the training and test set unchanged. We then applied the original models to these data, forecasting the change in CASP-12 score between waves 6 and 7 based on the predictors’ values at wave 6.

All models’ parameters, including the thresholds used to compute the accuracy of the logistic regression and LASSO, and the predictors that LASSO accounts for, were kept the same as those of the original models. AUC, accuracy and F1 score were computed and compared to the ones achieved with the original model. This approach tested the models on two sets of waves completely independent from those used to train the models, assessing their ability to predict the $\Delta {\text {CASP-12}}$ direction at a later stage than the one in which the models were trained.

#### Assessment of the stability of selected variables

Finally, to evaluate the stability of the most relevant predictors of CASP-12 score over time, we trained three entirely new models using the same set of subjects and predictors as presented in Table 1. In particular, we considered the data collected at waves 6 and 7 rather than waves 4 and 5 as in the original models, and for each modeling technique employed we trained a new model that predicted the CASP-12 variation between waves 6 and 7. To evaluate the consistency of the most important variables over time, we compared the variables selected by the new models at waves 6 and 7 with those identified by the original models at waves 4 and 5. We consider the predictors to be stable over time if the variables were consistently identified as relevant by both the new and original models.

## Results

### Preprocessing

A total of 9422 subjects met the inclusion criteria, of whom 7584 were assigned to the training set and 1838 to the test set. Using a threshold of 0 to discretise the outcome, we obtained two balanced classes: in the training set, 4318 subjects belonged to class 0 ($\Delta {\text {CASP-12}}\ge 0$) and 3266 to class 1 ($\Delta {\text {CASP-12}}<0$), while in the test set, 1029 subjects belonged to class 0 and 809 to class 1.

### Models of CASP-12 variation

For the LASSO regularisation, $\lambda$ values in the range [0.0006874, 0.1381176] were tested. The optimal value of $\lambda$ was found to be 0.0028. The LR model and LASSO identified the same optimal threshold of 0.44 for assigning probabilities to predicted classes. Table 2 presents the coefficients estimated with LR, the corresponding *p*-values, and the coefficients obtained with the LASSO model. The latter retained 17 out of 21 predictors, discarding the remaining four due to their low contribution to the model. 500 decision trees were trained to build the random forest, as there was no significant improvement in performance by increasing the number of trees; the optimal value of the considered variables at each step *m* (as defined in Appendix II, Additional file 1) resulted to be 4.

**Table 2:** LR model coefficients with respective *p*-values, and LASSO model coefficients, for models predicting CASP-12 difference between Wave 5 and Wave 4 using data from Wave 4

Variable	LR model		LASSO model coefficient
	coefficient	*p*-value	
Intercept	-4.766	<0.001	-4.448
Age	0.301	0.086	0.261
Gender, male	0.007	0.899	0
ISCED	-0.299	0.011	-0.283
CASP-12, wave 4	6.156	<0.001	5.809
Cars	-0.213	0.018	-0.172
Ends meet	-0.796	<0.001	-0.727
Owner-occupier, owner	-0.796	<0.001	-0.727
Health	0.820	<0.001	0.795
Mobility	0.242	0.172	0.198
ADL	-0.311	0.363	0
IADL	0.940	0.009	0.704
Vigorous activities	0.113	0.076	0.097
Moderate activities	0.047	0.615	0.017
EURO-D	0.821	<0.001	0.667
Left out	0.088	0.388	0.077
Social activities	-0.625	<0.001	-0.594
Marital status, living together	0.066	0.262	0.014
Marital status, never married	0.046	0.610	0
Current job, retired	0.035	0.632	0.024
Current job, unemployed	0.028	0.798	0
Chronic diseases	0.097	0.608	0.051

**Fig. 2:**
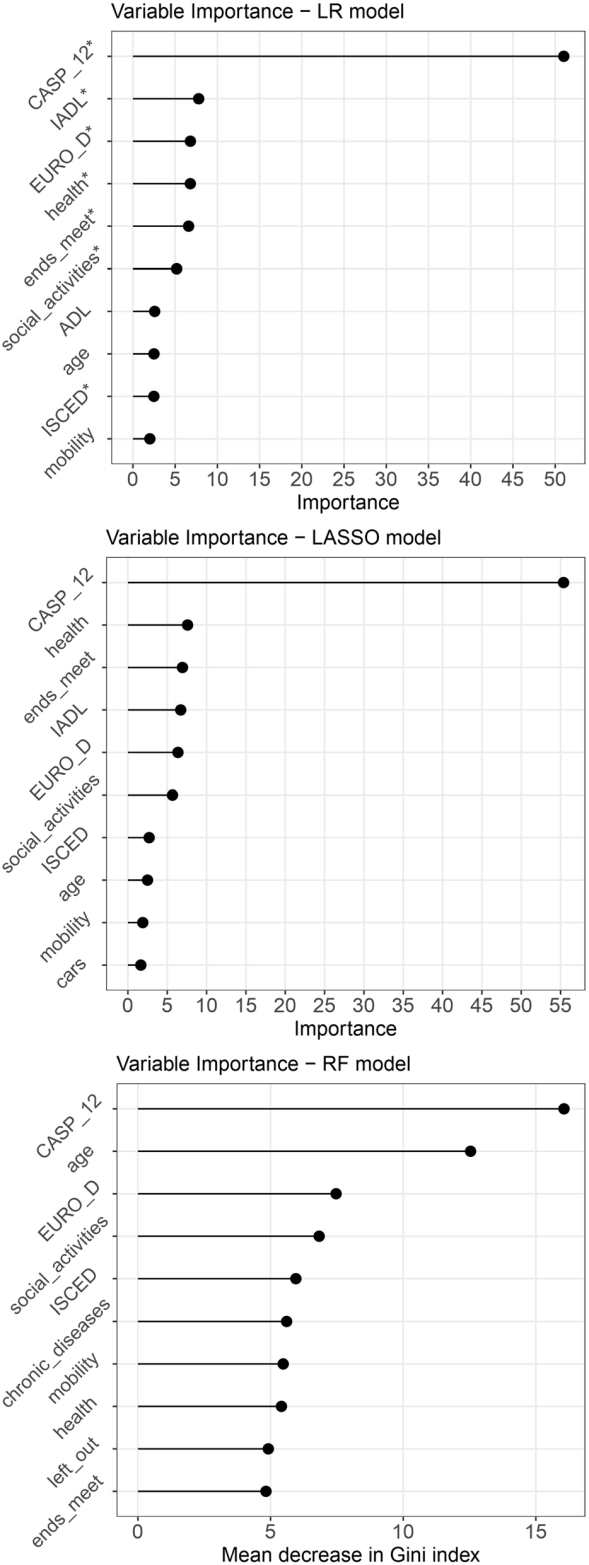
Features ranking for LR, LASSO, and RF models, trained on data from the fourth wave to forecast CASP-12 variation between the 4^th^ and the 5^th^ wave. X-axis is a scale based on the absolute value of the coefficients for LR and LASSO and based on the mean decrease in Gini index for the RF model. On the y-axis, the names of the variables are displayed. The predictors that in Table 2 have a *p*-value <0.05 are highlighted with a $*$ next to the variable name

In Fig. 2, we represent the top ten predictors for LR and LASSO models, ordered by the absolute value of their coefficient. We consider the most relevant those with a greater coefficient. The sign of each coefficient, reported in Table 2, must be taken into account while interpreting the results because a strong predictor with a positive sign will increase the predicted probability, while a strong predictor with a negative sign will, on the opposite, lower it. The two methods display similar results, with the CASP-12 score at baseline being the strongest predictor (contributing at 51% for LR and 53% for LASSO). The predictors with a *p*-value < 0.05 for the LR model are IADL, EURO-D score, health, ends meet, social activities, and ISCED, and they appear to be the strongest predictors also in the LASSO model, albeit in a slightly different order.

The bottom plot in Fig. 2 reports on the y-axes the 10 most relevant variables for the RF model, according to the mean decrease in the Gini index. The x-axis represents the relative contribution of each variable to the RF model’s prediction: it ranges from 0 to 100, with the sum of the values of all the variables equal to 100. The contribution of predictors in the RF model resulted much more balanced than in the other models, with CASP-12 at baseline remaining the strongest predictor, followed by age, EURO-D score, number of social activities, and education level.

**Fig. 3:**
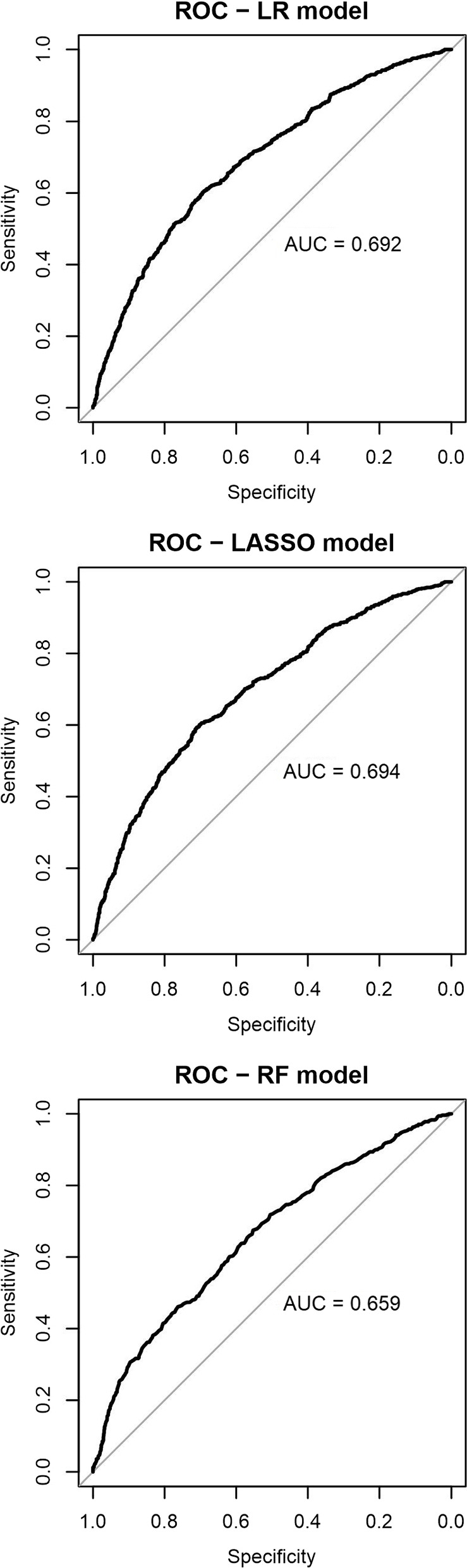
Receiver Operating Curve for the model developed with logistic regression, LASSO regularisation and random forest, trained on data from the fourth wave to predict CASP-12 variation between the 4^th^ and the 5^th^ wave

The LR and LASSO models achieved similar results, with an accuracy of 64%, AUC of 0.69, and an F1 score of 0.66. The RF model had a slightly higher accuracy of 65%, with an AUC of 0.66 and an F1 score of 0.71. Figure 3 shows the ROC curves for the models. For a more comprehensive overview of the results, Table 3 displays the confusion matrices for the LR, LASSO, and RF models. Both the LR and LASSO models show similar performance, achieving moderate accuracy in identifying the majority and minority classes. However, they demonstrate a certain number of false negatives and false positives. In contrast, the RF model exhibits higher accuracy in correctly identifying the majority class but encounters difficulties in identifying the observations belonging to the minority class, leading to an increased number of both false negatives and false positives.

**Table 3:** LR, LASSO and RF models confusion matrices for forecasting the CASP-12 difference between the 4^th^ and the 5^th^ wave, using Wave 4 as the baseline

Model	Predicted	Actual	
		**0**	**1**
LR model	0	656	373
	1	295	514
LASSO model	0	655	374
	1	295	514
RF model	0	795	234
	1	414	395

### Assessment of models’ generalisability and stability

#### Assessment of model’s generalisability on different subjects

The evaluation metrics (accuracy, AUC, and F1 score) are computed for each of the 10 tested training-test splits, and the mean and standard deviation of the metrics across all splits are reported in Table 4 to summarise the models’ general performance. In the first column, it is specified the metric considered, and for each metric is reported a row for the training set’s performance and a row for the test set’s performance. The last three columns report the performance of the LR model, LASSO model, and RF model respectively. The small standard deviation of the performance metrics across the different training-test splits indicates that the reported performance in Results section is robust and not dependent on the specific choice of training and test sets.

**Table 4:** Comparison of LR, LASSO, and RF models for predicting CASP-12 difference between Wave 5 and Wave 4 using data from Wave 4 as a baseline. Mean (sd) performance was computed on 10 different training and test set splits

Metric	Dataset	LR	LASSO	RF
accuracy	training set	65% (0.142)	65% (0.199)	65% (0.003)
	test set	64% (0.581)	64% (0.56)	64% (0.799)
AUC	training set	0.651 (0.001)	0.650 (0.002)	1 (0)
	test set	0.640 (0.006)	0.639 (0.006)	0.636 (0.01)
F1 score	training set	0.682 (0.002)	0.681 (0.002)	1 (0)
	test set	0.668 (0.006)	0.667 (0.006)	0.706 (0.007)

#### Assessment of models’ stability over time

To assess the stability of the models trained on waves 4 and 5, their performance was evaluated on waves 6 and 7. The same 1838 subjects were used for this analysis as in the original models, considering wave 6 as the baseline wave and $\Delta$CASP-12 between waves 7 and 6 as the outcome. Of these, 976 experienced an increase or no change in their CASP-12 scores ($\Delta {\text {CASP-12}}\ge 0$), while 862 had a decrease in their scores over time ($\Delta {\text {CASP-12}}<0$).

The LR model achieved an AUC of 0.610, an accuracy of 61%, and an F1 score of 0.630 on this test set. The LASSO model resulted in an AUC of 0.614, an accuracy of 61%, and an F1 score of 0.629. The RF model had an AUC of 0.608, an accuracy of 61%, and an F1 score of 0.667. Overall, the models performed about 3-4% lower on the later and independent waves compared to the original waves they were trained on, as reported in the second row of Table 5.

**Table 5:** Comparison of LR, LASSO, and RF models performance on the test set in different scenarios, assessing models’ stability

Scenario	Metric	Method		
		LR	LASSO	RF
Main model	accuracy	64%	64%	65%
	AUC	0.692	0.694	0.659
	F1 score	0.663	0.662	0.710
Models’ stability	accuracy	61%	61%	61%
	AUC	0.610	0.614	0.608
	F1 score	0.630	0.629	0.667
Variables’ stability	accuracy	63%	63%	62%
	AUC	0.629	0.630	0..615
	F1 score	0.649	0.645	0.659

#### Assessment of the stability of selected variables

Finally, three new models were trained with LR, LASSO regularisation and RF considering the predictors’ values at wave 6 to predict the binary outcome related to the variation of CASP-12 score between wave 6 and 7.

**Fig. 4:**
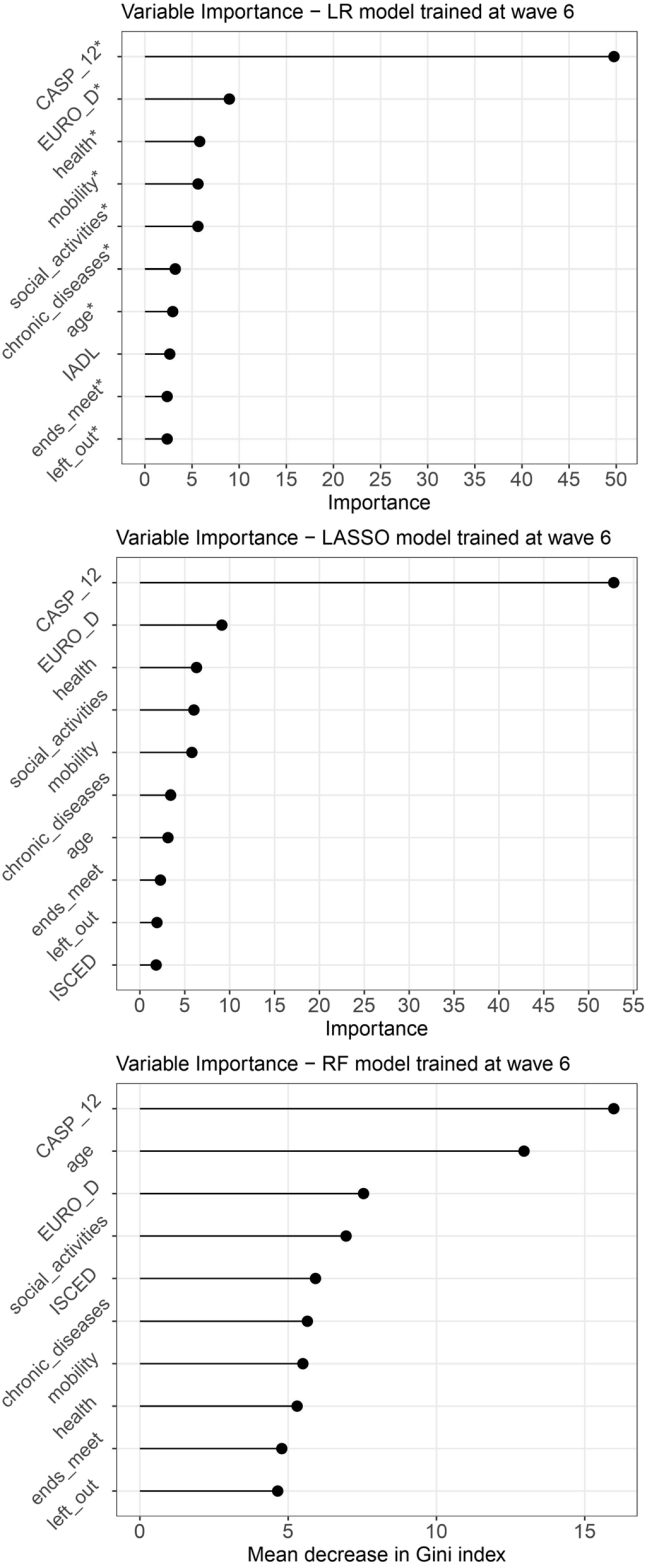
Features ranking for LR, LASSO, and RF models, trained on data from the sixth wave to forecast CASP-12 variation between the 6^th^ and the 7^th^ wave. X-axis is a scale based on the absolute value of the coefficients for LR and LASSO and based on the mean decrease in Gini index for the RF model. On the y-axis the names of the variables are displayed. The predictors that in Table 6 have a *p*-value <0.05 are highlighted with a $*$ next to the variable name

The LR model has AUC$=0.629$, accuracy $=63\%$, and F1 score $=0.649$. For the LASSO model, the $\lambda$ values tested were in the range [0.0006356598, 0.1163727] and the optimal value $\lambda _{opt}$ resulted to be 0.0017. Table 6 presents the coefficients estimated with LR, the corresponding *p*-values, and the coefficients obtained with the LASSO model. The obtained LASSO model reached AUC$=0.630$, accuracy$=63\%$, and F1 score$=0.645$, keeping all the variables available in the model. 500 decision trees were employed for the RF model (parameter *ntree*), while the optimal number of considered variables at each step (parameter *m*) resulted to be 5. The AUC of this model was 0.615, the reached accuracy was $=62\%$, and the F1 score was 0.659. Table 5 reports, on the third row, the presented performance and Fig. 4 reports the variable importance graphs for the three new models. The LR and LASSO plots are very similar, reporting the following variables among the most important ones, in the following order: CASP-12 value at baseline, EURO-D score, subjective assessment of health status, physical limitations in mobility and the number of social activities in which a subject is involved. CASP-12 value at baseline is also the strongest predictor for the RF model, together with the subjects’ age. These are followed by EURO-D score, number of social activities, and level of instruction.

**Table 6:** LR model coefficients with respective *p*-values, and LASSO model coefficients, for models predicting CASP-12 difference between Wave 7 and Wave 6 using data from Wave 6

Variable	LR model		LASSO model coefficient
	coefficient	*p*-value	
Intercept	-4.727	<0.001	-4.51
Age	0.355	0.035	0.334
Gender, male	0.166	0.003	0.136
ISCED	-0.212	0.062	-0.193
CASP-12, wave 4	5.989	<0.001	5.645
Cars	-0.15	0.094	-0.133
Ends meet	-0.284	0.001	-0.244
Owner-occupier, owner	0.003	0.96	0
Health	0.699	<0.001	0.674
Mobility	0.678	<0.001	0.619
ADL	0.278	0.337	0.13
IADL	-0.317	0.293	-0.04
Vigorous activities	0.128	0.041	0.119
Moderate activities	0.17	0.053	0.14
EURO-D	1.078	<0.001	0.976
Left out	-0.284	0.007	-0.203
Social activities	-0.677	<0.001	-0.643
Marital status, living together	-0.023	0.693	-0.012
Marital status, never married	0.007	0.934	0
Current job, retired	0.063	0.405	0.043
Current job, unemployed	0.088	0.463	0.043
Chronic diseases	0.387	0.032	0.366

When comparing the plots in Fig. 4 with those obtained from the models trained on the fourth wave (Fig. 2), it becomes apparent that, besides the CASP-12 score, certain variables consistently show high importance regardless of the method used. Specifically, the EURO-D score related to depression and the number of social activities are consistently relevant for all models, whereas the variable related to economic difficulties (i.e. *endsmeet*) that was significant for the original models trained on waves 4-5, does not have the same relevance for the models trained at a later stage. Additionally, the contribution of age is emphasised in both RF models, while the regression models do not place as much emphasis on this variable.

## Discussion

The aim of this study was to explore the feasibility of developing a forecasting model that could distinguish individuals who experience a 2-year decline in perceived wellbeing, as measured by the CASP-12 score, using a subset of demographic, social, health-related, and financial variables. Understanding the variables that contribute to changes in subjective wellbeing is important for promoting healthy ageing: by identifying individuals who may be at risk for a decline in subjective wellbeing, this study may contribute to the development of targeted interventions to prevent adverse outcomes, such as the onset of chronic diseases [10, 11, 13].

One notable strength of this study is the use of the SHARE dataset, a unique panel database of micro data on health, social, and economic status, covering most of the European Union and Israel. Freely available to the scientific community, with its longitudinal nature and over 150,000 interviews, provides a comprehensive picture of life after the age of 50 and allows for the exploration of ageing population characteristics, such as perceived wellbeing. Additionally, the SHARE study is harmonised with other longitudinal studies on ageing, such as the ELSA, making it a role model for ageing surveys worldwide.

The forecasting models developed in this study, along with the most relevant variables, were shown to be reliable, as their generalisability was assessed on different subjects and time windows. The absence of overfitting was confirmed, and the models’ stability and applicability over time were demonstrated, with only a 3-4% decrease in accuracy after four years.

The stability of selected variables was also assessed to understand whether the strongest predictors of a variation in CASP-12 score were well-defined or could vary according to the training set. The baseline CASP-12 score was consistently the strongest predictor across all methods. Depression score, the number of social activities, and difficulties in making ends meet were also frequently among the most relevant variables, and have been reported to be related to the wellbeing of the population over 50 in previous studies [8, 14]. Age at baseline was highly relevant in models trained with the RF method but not in LR and LASSO. However, it is important to note that all models found that a subject is more likely to experience a decrease in perceived wellbeing if they have a low CASP-12 score, perceived poor general health, high EURO-D score (severe depression), scarce participation in social activities, and advanced age. Additional models were trained including as a predictor the variable reporting the subjects’ country of residence. The performance of these models was slightly better than that of the original models ($\sim$1% higher accuracy), but the inclusion of this variable would have limited their applicability, thus they are not reported in detail.

To the best of our knowledge, only descriptive models are presented in the literature, therefore this is the first study assessing the possibility of forecasting ahead of time the worsening of subjective perception of well-being as measured by the CASP-12 score.

Despite the strengths of this study, there are also some critical aspects and limitations to consider. One critical point is the lack, in the literature, of a “Minimal Clinically Important Difference” [21] for $\Delta$CASP-12. For this reason, we decided to categorise the outcome, accounting for any decrease/not decrease of CASP-12, instead of considering another arbitrary minimal variation. Should a threshold for defining minimal clinically relevant variations of CASP-12 become available in the future, the models developed in this work could be updated to take this into account.

The subject selection process, as described in Fig. 1, may constitute a limitation of the study as any reduction creates a slightly different dataset, potentially introducing bias. To account for this, statistical tests have been applied on the variables’ distribution, and their results are presented in Table 1. Although we acknowledge that some variables’ distributions are statistically different, most of the variables’ distributions are similar over the selection process. This indicates that the final dataset, composed of 9422 subjects, can be considered a well-representative sample of the original one. As future developments, we aim to address this issue by including subjects with missing data.

The subjective nature of the CASP-12 score, being a self-reported measure of wellbeing obtained by questionnaire, may also limit the performance of predictive models, as it is potentially noisy and unstable. Moreover, self-reported measures have many limits, such as comparability across countries, across individuals, and over time, and omitting or unmeasured confounders, such as personality traits and attitudes towards life, may represent more relevant reasons for the limited performance of the model.

As Ward points out, the trajectories of the CASP-12 score over time are highly diverse among individuals, as the score reflects various aspects of an older person’s life, including both objective and subjective parameters [22]. Thus, developing a forecasting and population-based model of the variation of the score is challenging.

In conclusion, this study provides insights into the variables that affect subjective wellbeing in the elderly population, with the developed models and most relevant variables demonstrating reliability and stability over time. However, the subjective nature of the CASP-12 score and the diverse trajectories of the score among individuals pose limitations to the development of predictive models, highlighting the need for continued research in this area.

## Conclusion

In this study, we investigated the possibility of predicting the change in subjective wellbeing over time in the ageing population using machine learning models fed by demographic, health, social, and financial variables. Although the models’ performance was limited, we demonstrated their stability across different subjects and over time. Additionally, we identified the most relevant predictors of the 2-year variation in CASP-12 scores, shedding light on the variables that influence subjective wellbeing in the elderly population. To our knowledge, this is the first study to use a purely longitudinal approach to identify predictors of subjective wellbeing in the elderly population, and our findings could inform the development of targeted interventions to promote healthy ageing.

Future developments may include assessing the economic consequences of the study to inform social policies targeting the identified predictors, predicting CASP-12 variations over a longer prediction horizon, developing more complex models or including a larger set of variables to stabilise the models in time, and testing the models on different populations or including subjects with missing variables. These future directions will allow us to gain a deeper understanding of the predictors of subjective wellbeing in the ageing population.

### Supplementary Information

**Additional file 1.**

## Data Availability

The datasets that support the findings of this study are available, upon registration, at http://www.share-project.org/data-documentation/share-data-releases.html with the identifiers “Wave 4”, “Wave 5”, “Wave 6”, and “Wave 7” [DOIs: “10.6103/SHARE.w4.800”, “10.6103/SHARE.w5.800”, “10.6103/SHARE.w6.800”, “10.6103/SHARE.w7.800”]. The R code needed to reproduce the results presented can be accessed in the following public GitHub repository: https://github.com/IsottaT/wellbeing_forecasting_models.
