# Supplementary material for: A model to forecast the two-year variation of subjective wellbeing in the elderly population
Source: BMC Med Inform Decis Mak. 2023 Nov 8;23:253. doi: 10.1186/s12911-023-02360-8 (PMC10634107; doi:10.1186/s12911-023-02360-8)
Supplement: Supplementary file 1 — Additional file 1. [file 12911_2023_2360_MOESM1_ESM.pdf]

# Additional file 1

### Appendix I: Details on linear methods for the development of CASP-12 predictive model

A commonly used method for predicting binary outcomes is Logistic Regression (LR), which is regarded as a generalised linear method because it produces a linear boundary, although the response is not a linear function of the parameters. Specifically, given a set of $n$ predictors that may be numeric or categorical, logistic regression computes the probability of an entry belonging to a class through the logit model:

$$ln\Big(\frac{p}{1-p}\Big) = \beta_0 + \sum_{j=1}^{n} \beta_j X_j + \epsilon \quad (1)$$

where $p$ represents the probability that the event occurs, $\beta_0$ denotes the intercept, $\beta_j$ are the model coefficients, $X_j$ are the predictor variables, and $\epsilon$ refers to the model's error. This method generates a probability score between 0 and 1. Observations with probabilities greater than the threshold are classified as belonging to class 1, and those below the threshold are classified as belonging to class 0. The assignment threshold is determined by selecting the probability value that corresponds to the closest point to the upper left corner of the ROC curve.

A second logistic model with LASSO regularisation was fitted to reduce the number of variables. In the following, this model will be referred to as the LASSO model. The goal of the LASSO approach is to estimate the model coefficients $\hat{\beta}^{LASSO}$ by minimising the sum of the residual sum of squares (RSS) and the LASSO penalty term, which includes the regularisation parameter $\lambda$, as represented by Equation (2).

$$\hat{\beta}^{LASSO} = \underset{\beta}{argmin}\{RSS + \lambda \sum_{j=1}^{p} |\beta_j|\} \quad (2)$$

The optimal $\lambda$ value has been searched in the range $[\lambda_{min}, \lambda_{max}]$: $\lambda_{min}$ is set to $\lambda_{min} = 0.001 * \lambda_{max}$, and $\lambda_{max}$ is selected so that beta forces all parameter estimates to zero for $\lambda > \lambda_{max}$. Within this range, a grid of 100 equally spaced points on the logarithmic scale is considered and the optimal $\lambda$ value is the one producing the smallest deviance on 10-fold cross-validation performed on the training set [1].

We also tested the Elastic Net (EN) model which combines the L1 and L2 penalties to achieve both variable selection and shrinkage. The EN model estimates coefficients by minimising the residual sum of squares (RSS), the L1 penalty term for sparsity, and the L2 penalty term for small coefficient values. The regularisation parameters $\lambda$ and $\alpha$ control the strength of the penalties, with $\lambda$ determining the overall level of regularisation and $\alpha$ balancing the relative importance of the L1 and L2 penalties. When $\alpha = 1$, the EN model reduces

to the LASSO model, and when $\alpha = 0$, it reduces to Ridge Regression. To build the EN model, we used 10-fold cross-validation, searching in a grid with 25 $\alpha$ values and 25 $\lambda$ values at each repetition. However, we found that the model performances were not significantly different from those obtained with the LASSO model, and the parameter estimates were collapsing to those obtained with the LASSO model. Therefore, we proceeded with the LASSO model as it provided a simpler and more interpretable model.

### Appendix II: Details on non-linear method for the development of CASP-12 predictive model

Linear models have some limitations, such as the assumption of linearity between the input features and the outcome, which may not hold in real-world scenarios. Additionally, they may not be able to capture complex non-linear relationships in the data, which can lead to lower accuracy and performance. To overcome these possible limitations, other models have been tested with an easily interpretable, non-linear, classification algorithm such as the random forests (RF). RF are a non-linear, interpretable classification algorithm based on a set of de-correlated decision trees that operate as an ensemble. A decision tree can handle both categorical and numerical variables to assign an entry to a class, according to a decision rule learned on the training set. The final outcome is the one that most frequently occurred amongst all the trees, reducing the risk of overfitting and producing highly accurate predictions even with large datasets. To fit the RF model, we evaluated various combinations of the number of trees in the forest (*ntree*) and the number of variables at each split ($m$). Specifically, we considered all integer values of trees between 500 and 1000, and m ranging from 4 to 8. The optimal combination was determined by assessing the model's performance on 10-fold cross-validation performed on the training set and selecting the one that resulted in the best overall accuracy value. We also assessed feature importance by evaluating the mean decrease in the Gini index, a measure of total variance across the two classes in which the outcome is divided. The Gini index is defined as:

$$Gini = \sum_{i=1}^{K} \hat{p}_{mk}(1 - \hat{p}_{mk}) \tag{3}$$

where $\hat{p}_{mk}$ represents the proportion of observations in the $m$th node that are from the $k$th class. A small $\hat{p}_{mk}$ implies that a node contains predominantly observations from a single class, and this leads to a low Gini index, which is considered a measure of node purity [2].

# References


[1] Hastie, T. & Qian, J. Glmnet vignette. *Retrieved June.* **9**, 1-30 (2014)

[2] Sohil, F., Sohali, M. & Shabbir, J. An introduction to statistical learning with applications in R: by Gareth James, Daniela Witten, Trevor Hastie, and Robert Tibshirani, New York, Springer Science and Business Media, 2013, $41.98, eISBN: 978-1-4614-7137-7. *Statistical Theory And Related Fields.* pp. 1-1 (2021,9), https://www.tandfonline.com/doi/full/10.1080/24754269.2021.1980261
